# Polar angle asymmetries persist despite covert spatial attention and differential adaptation

**DOI:** 10.1101/2025.09.30.676904

**Published:** 2025-10-02

**Authors:** Hsing-Hao Lee, Marisa Carrasco

**Affiliations:** 1 Department of Psychology, New York University, New York, NY, USA; 2 Center for Neural Sciences, New York University, New York, NY, USA

**Keywords:** visual adaptation, endogenous attention, exogenous attention, visual performance asymmetries, contrast sensitivity

## Abstract

Visual adaptation and attention are two processes that help manage brain limited bioenergetic resources for perception. Visual perception is heterogeneous around the visual field, it is better along the horizontal than the vertical meridian (horizontal-vertical anisotropy, HVA), and better along the lower than the upper vertical meridian (vertical meridian asymmetry, VMA). A recent study showed that visual adaptation is more pronounced at the horizontal than the vertical meridian, but whether and how the differential adaptation modulates the effects of covert spatial attention remains unknown. In this study, we examined how the effects of endogenous (voluntary) and exogenous (involuntary) covert attention on an orientation discrimination task vary at the cardinal meridians, with and without adaptation. We manipulated endogenous (Experiment 1) or exogenous (Experiment 2) attention via a central or peripheral cue, respectively. Results showed that (1) in the non-adapted condition, the typical HVA and VMA emerged in contrast threshold; (2) the adaptation effect was stronger at the horizontal than the vertical meridian; and (3) regardless of adaptation, both endogenous and exogenous attention enhanced and impaired performance at the attended and unattended locations, respectively, to a similar degree at both cardinal meridians. These findings reveal that, despite a differential adaptation effect, performance asymmetries are resistant to both endogenous and exogenous attention around polar angle.

## Introduction

Visual adaptation and attention are two processes that can help manage the limited bioenergetic resources in the brain and allocate them according to the task demand (Carrasco, 2011; Lee, Fernández, & Carrasco, 2024; Lennie, 2003; Pestilli, Viera, & Carrasco, 2007). Visual adaptation reduces sensitivity toward repeated features and enhances sensitivity toward novel ones (Boynton & Finney, 2003; Gardner et al., 2005; Kohn, 2007; Perini, Cattaneo, Carrasco, & Schwarzbach, 2012; Vergeer, Mesik, Baek, Wilmerding, & Engel, 2018; Webster, 2011, 2015); visual attention selectively improves information processing at a given locations while impairing processing elsewhere (e.g., Ling & Carrasco, 2006a; Lu, Lesmes, & Dosher, 2002; Pestilli & Carrasco, 2005; Pestilli, Ling, & Carrasco, 2009; Pestilli et al., 2007), for reviews (Carrasco, 2011, 2014; Olivers, 2025)

There are two types of covert spatial attention: endogenous and exogenous. Endogenous attention is voluntary, goal-driven, flexible, and sustained, whereas exogenous attention is involuntary, stimulus-driven, automatic, and transient (reviews: Carrasco, 2011, 2014; Olivers, 2025). These two types of covert attention share similar effects on numerous visual tasks, e.g., contrast sensitivity (e.g., Herrmann, Montaser-Kouhsari, Carrasco, & Heeger, 2010; Pestilli et al., 2009), appearance (reviews: Carrasco & Barbot, 2019), and orientation discrimination (e.g., Fernández, Okun, & Carrasco, 2022), but have distinct effects in other tasks, e.g., texture segregation (e.g., Barbot & Carrasco, 2017; Jigo, Heeger, & Carrasco, 2021; Yeshurun & Carrasco, 1998), and alter sensitivity in a different spatial frequency range (Fernández et al., 2022; Jigo & Carrasco, 2020).

**Figure 1.**
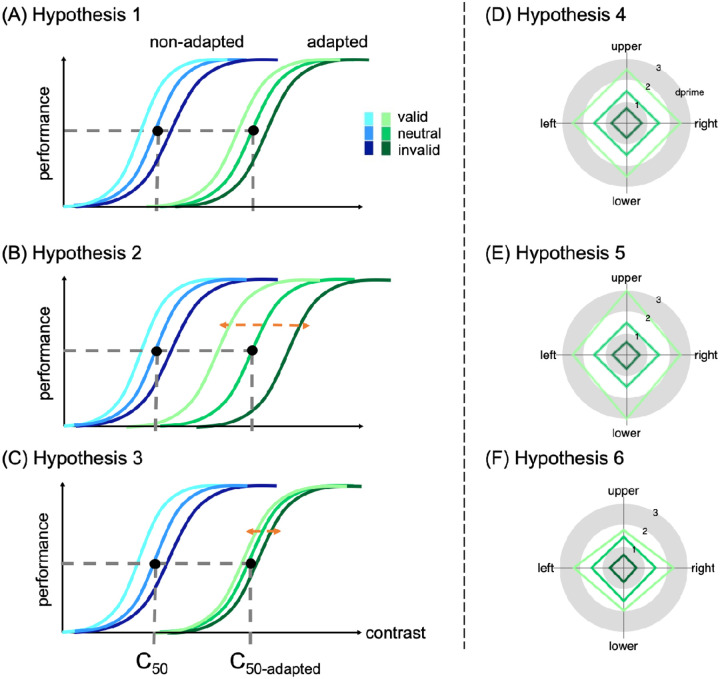
Possible hypotheses. (A) Hypothesis 1: Attentional effect is comparable with and without adaptation. The c_50_ and c_50_-adapted indicate the contrast threshold derived from the titration procedures in the non-adapted and adapted conditions, respectively. (B) Hypothesis 2: Attentional effect is larger with than without adaptation. (C) Hypothesis 3: Attentional effect is smaller than without adaptation. (D) Hypothesis 4: Attentional effect is comparable around polar angle after adaptation. (E) Hypothesis 5: Attentional effect is stronger at the vertical than horizontal meridian. (F) Hypothesis 6: Attentional effect is smaller at the vertical than horizontal meridian.

Both adaptation (Dao, Lu, & Dosher, 2006; Gardner et al., 2005; Perini et al., 2012; Pestilli et al., 2007) and endogenous attention (Ling & Carrasco, 2006a; Pestilli et al., 2009) primarily affect the contrast gain of the contrast response function (Figure 1A), i.e., a shift in threshold, whereas exogenous attention primarily affects response gain (Fernández & Carrasco, 2020; Pestilli et al., 2009), i.e., a shift in asymptote. Additionally, according to a prominent normalization model of attention (Reynolds & Heeger, 2009), exogenous attention can also affect contrast gain when the attentional window is wide enough, whereas endogenous attention can also affect response gain when the attentional window is narrow (Herrmann et al., 2010).

Interestingly, exogenous attention still restores contrast sensitivity after adaptation (Lee et al., 2024; Pestilli et al., 2007). However, it is unknown whether and how endogenous attention restores contrast sensitivity after adaptation. It is possible that after adaptation endogenous attention enhances contrast sensitivity (1) similar to its effect before adaptation (Figure 1A; Hypothesis 1), given that endogenous attention and adaptation may not interact with each other and thus yield independent effects on contrast sensitivity; (2) more than before adaptation (Figure 1B; Hypothesis 2), in a compensatory manner, given the flexible nature of endogenous attention, which optimizes performance as a function of task demands (Barbot & Carrasco, 2017; Barbot, Landy, & Carrasco, 2012; Giordano, McElree, & Carrasco, 2009; Hein, Rolke, & Ulrich, 2006; Yeshurun, Montagna, & Carrasco, 2008); or (3) less than the effect before adaptation (Figure 1C; Hypothesis 3); given that adaptation compresses the contrast response function (Dao et al., 2006; Gardner et al., 2005; Perini et al., 2012; Vinke, Bloem, & Ling, 2022), endogenous attention may have limited push-pull effects on contrast sensitivity. Thus, the first goal of the current study is to examine whether adaptation modulates the effect of endogenous attention on contrast sensitivity.

Does target location matters? Visual performance is better along the horizontal than vertical meridian (horizontal-vertical anisotropy, HVA), and along the lower vertical than upper vertical meridian (vertical meridian asymmetry, VMA). These visual field asymmetries, known as performance fields, are present in numerous visual domains, including contrast sensitivity (Abrams, Nizam, & Carrasco, 2012; Baldwin, Meese, & Baker, 2012; Cameron, Tai, & Carrasco, 2002; Carrasco, Talgar, & Cameron, 2001; Corbett & Carrasco, 2011; Fuller, Rodriguez, & Carrasco, 2008; Himmelberg, Winawer, & Carrasco, 2020; Lee & Carrasco, 2025; Purokayastha, Roberts, & Carrasco, 2021), visual acuity (Kwak, Hanning, & Carrasco, 2023; Montaser-Kouhsari & Carrasco, 2009), spatial resolution (Altpeter, Mackeben, & Trauzettel-Klosinski, 2000; Carrasco, Williams, & Yeshurun, 2002; Greenwood, Szinte, Sayim, & Cavanagh, 2017; Talgar & Carrasco, 2002), motion (Fuller & Carrasco, 2009; Tünçok, Kiorpes, & Carrasco, 2025), as well in mid-level visual processing, including texture segregation (Barbot, Xue, & Carrasco, 2021; Greenwood et al., 2017; Talgar & Carrasco, 2002; Z. Wang, Murai, & Whitney, 2020), crowding (Greenwood et al., 2017; Kurzawski et al., 2023; Petrov & Meleshkevich, 2011), visual short-term memory (Montaser-Kouhsari & Carrasco, 2009), and higher cognitive task, such as numerosity perception (Chakravarthi, Papadaki, & Krajnik, 2022), face perception (Afraz, Pashkam, & Cavanagh, 2010; Kim & Chong, 2024), and word identification (Tsai, Liao, Hou, Jang, & Chen, 2024).

These visual field asymmetries are resistant to the effects of both endogenous (Purokayastha et al., 2021; Tünçok, Carrasco, & Winawer, in press) and exogenous attention (Cameron et al., 2002; Carrasco et al., 2001; Roberts, Ashinoff, Castellanos, & Carrasco, 2018; Roberts, Cymerman, Smith, Kiorpes, & Carrasco, 2016), and temporal (Fernández, Denison, & Carrasco, 2019) attention. Thus, the performance field is not easily reshaped. On the contrary, presaccadic attention, a type of overt attention that enhances the processing of the target location right before a saccade occurs, can exacerbate the performance asymmetries (Hanning, Himmelberg, & Carrasco, 2022, 2024; Kwak, Hanning, & Carrasco, in press; Kwak, Zhao, Lu, Hanning, & Carrasco, 2024). Whether adaptation modulates the uniform effect of covert attention on performance remains an open question.

A recent study showed that visual adaptation is stronger at the horizontal than the vertical meridian, leading to more homogeneous perception by mitigating the HVA (Lee & Carrasco, 2025). It remains unknown, however, whether and how endogenous and exogenous attention reshape the performance field after such differential adaptations. Following adaptation, covert spatial attention enhances contrast sensitivity (1) to the same extent around polar angle (Figure 1D; Hypothesis 4), similar to without adaptation (e.g., Carrasco et al., 2001; Purokayastha et al., 2021; Roberts et al., 2018; Roberts et al., 2016; Tünçok et al., in press). (2) more in the vertical than the horizontal meridian, and more in the upper than the lower vertical meridian, acting as a compensatory mechanism to mitigate asymmetries (Figure 1E; Hypothesis 5), or (3) more where performance is better (i.e., the horizontal meridian) than where it is worse (i.e., vertical meridian, especially the upper vertical meridian) (Figure 1F; Hypothesis 6).

Further, because endogenous attention is flexible (Barbot & Carrasco, 2017; Barbot et al., 2012; Giordano et al., 2009; Hein et al., 2006; Yeshurun et al., 2008), but exogenous attention is not (Barbot et al., 2012; Carrasco, Loula, & Ho, 2006; Crotty, Massa, Tellez, White, & Grubb, 2025; Giordano et al., 2009; Hein et al., 2006; Luck & Thomas, 1999; Yantis & Jonides, 1996; Yeshurun & Carrasco, 1998), and because different brain regions are critical for their effect – right frontal eye fields for endogenous attention (Fernández, Hanning, & Carrasco, 2023), whereas the early visual cortex for exogenous attention (Fernández & Carrasco, 2020; Lee et al., 2024), where it interacts with adaptation (Lee et al., 2024)– it is possible that endogenous and exogenous attention exert different effects on contrast sensitivity around polar angle after differential adaptation effects. Therefore, another goal of the current study is to examine if endogenous and exogenous attention have different effects on contrast sensitivity after visual adaptation.

In brief, we asked (1) whether and how endogenous and exogenous attention restore contrast sensitivity before and after adaptation, (2) whether endogenous and exogenous attention restore contrast sensitivity to a similar extent around the visual field before and after adaptation. These findings are critical to unveil how we allocate resources according to task demands through adaptation and attention in tandem—two fundamental visual processes that help manage limited bioenergetic resources.

## Experiment 1

### Methods

#### Participants

Twelve adults (5 females, age range: 24–36 years old), including author HHL, participated in the experiment. All of them had normal or corrected-to-normal vision. Sample size was based on previous studies on adaptation (Lee et al., 2024), with an effect size of *d*=1.3, and on performance fields (Lee & Carrasco, 2025), with an effect size of *d*=1.41 for performance in the neutral trials. According to G*Power 3.0 (Faul, Erdfelder, Lang, & Buchner, 2007), we would need 9 participants for adaptation and 8 participants for performance fields to reach a power=0.9. We also estimated the required sample size for the interaction between adaptation and location, based on a recent study between adaptation and performance fields (Lee & Carrasco, 2025) (*η*_*p*_^*2*^=0.34), by assuming SD=1, we would need 10 subjects to reach a power=0.9 according to the Monte-Carlo simulation (1,000 iterations per possible subject number). The Institutional Review Board at New York University approved the experimental procedures, and all participants provided informed consent before they started the experiment.

#### Stimuli and apparatus

The target Gabor (diameter = 4°, 5 cpd, 1.25° full-width at half maximum) was presented on the left, right, upper and lower cardinal meridian locations (8° from the center to center). There were four placeholders (length = 0.16°, width = 0.06°) 0.5° away from the Gabor’s edge. The fixation cross consisted of a plus sign (length = 0.25°; width = 0.06°) at the center of the screen. The endogenous attentional cue (length = 0.75°; width = 0.2°) was presented at the center.

Participants were in a dimly lit, sound-attenuated room, with their head placed on a chinrest 57 cm away from the monitor. All stimuli were generated using MATLAB (MathWorks, MA, USA) and the Psychophysics Toolbox (Brainard, 1997; Pelli, 1997) on a gamma-corrected 20-inch ViewSonic G220fb CRT monitor with a spatial resolution of 1,280 × 960 pixels and a refresh rate of 100 Hz. To ensure fixation, participants’ eye movements were recorded using EYELINK 1000 (SR Research, Osgoode, Ontario, Canada) with a sample rate of 1,000 Hz.

#### Experimental design and procedures

**Figure 2.**
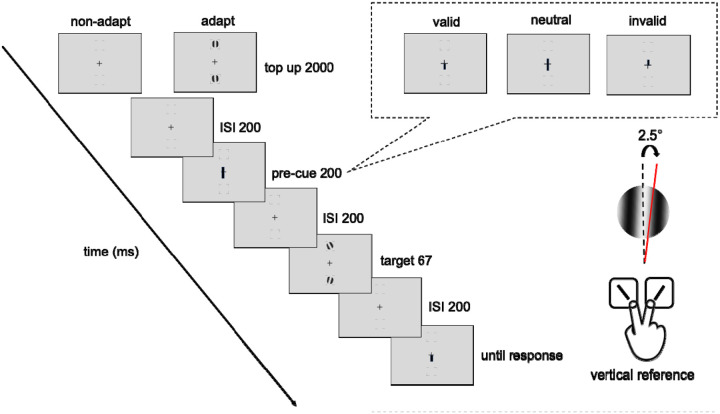
Experimental procedure: Participants performed either adaptation or nonadaptation blocks, each in separate experimental sessions. The target Gabor stimulus was always presented within the black placeholder, and target meridians were blocked. The target, two vertical Gabor stimuli were presented 8° away from the center. Participants were instructed to respond whether the Gabor was tilted clockwise or counterclockwise from vertical. The pre-cue either matches (valid condition), mismatches (invalid condition) the response cue, or does not provide location information (neutral condition). For illustration purposes, the stimulus size and spatial frequency shown here are not to scale.

Figure 2 shows the procedure of titration and the endogenous attention task. In the adapted condition, at the beginning of each block, participants adapted to a vertical 5-cpd Gabor patch flickering at 7.5 Hz in a counterphase manner, presented at the target location for 60 seconds. Each trial started with a 2s top-up phase to ensure a continuous adaptation effect throughout the block. In the non-adaptation condition, participants maintained fixation at the center for 4s (without Gabor) at the beginning of each block and for 2s at the beginning of each trial.

After the top-up, there was a 200 ms ISI before an endogenous pre-cue was presented for 100 ms. Following a 200-ms ISI the tilted Gabor was then presented for 67 ms, followed by another 200-ms ISI and then the response cue. In a valid trial, the location indicated by the response cue matches the precue; in an invalid trial, they mismatch; in a neutral cue condition, the precue points at both locations. Participants had to judge whether the target Gabor was tilted clockwise or counterclockwise off vertical. The tilt angle was 2.5°, based on pilot data and our previous study (Lee & Carrasco, 2025), to ensure an adaptation effect while avoiding floor or ceiling performance.

A feedback tone was presented when participants gave an incorrect response. The target locations were blocked in a horizontal block or a vertical block, where the target locations were presented at the horizontal or vertical meridians, respectively. Participants were asked to respond as accurately as possible while fixating at the center of the screen throughout the trial. A trial would be interrupted and repeated at the end of the block if participants’ eyes position deviated ≥1.5° from the center, from the pre-cue onset until the response cue onset.

Participants completed the adapted and non-adapted attentional task on the vertical and the horizontal meridian on different days, with a counterbalanced order. The order of horizontal and vertical meridian blocks was randomized, and the adaptation and non-adaptation titration were implemented on different days, with a counterbalanced order. There were 4 independent staircases for each adaptation condition and location, varying Gabor contrast from 2% to 85% to reach ~75% accuracy for the orientation discrimination task. Each staircase started from 4 different points (85%, 2%, the median contrast of 43.5%, and a random point between 2% and 85%) and contained 48 trials. Four blocks (192 trials per location for each adaptation and nonadaptation conditions) were conducted consecutively for the horizontal meridian block or the vertical meridian block. The contrast threshold was derived using an adaptive staircase procedure using the Palamedes toolbox (Prins & Kingdom, 2018), as in previous studies (e.g., Fernández & Carrasco, 2020; Hanning et al., 2022; Jigo & Carrasco, 2018; Lee & Carrasco, 2025; Lee et al., 2024) and averaging the last 8 trials. The Gabors were always preceded by a neutral pre-cue which provided no location information regarding the target.

In this endogenous attention task, for each adapted and non-adapted condition, 20% of the trials had a neutral cue, which pointed at both locations; 80% of the trials had an attentional cue pointing toward a location, 75% among them were valid cues, and the other 25% were invalid cues. All participants completed a practice session to familiarize themselves with the task procedure.

#### Psychometric function fitting

We fitted a Weibull function for the accuracy as a function of contrast threshold. For each location and adaptation condition, a logistic function was fit to the data using maximum likelihood estimation using the fmincon function in MATLAB. The results derived from the psychometric function estimation positively correlated (*ps*<.01) with the staircase results in all experiments, verifying our procedure in all conditions.

#### Behavioral data analyses

Behavioral data analyses were performed using R (Team, 2000). A three-way repeated-measures analysis of variance (ANOVA) on d’ was conducted on the factors of location (horizontal meridian, upper, lower), adaptation (adapted, non-adapted), and attention (valid, neutral, invalid) conditions to assess statistical significance. Repeated-measures ANOVA along with effect size (*η*^*2*^) were computed in R and used to assess statistical significance.

### Results

#### Adaptation effect varied around polar angle

**Figure 3.**
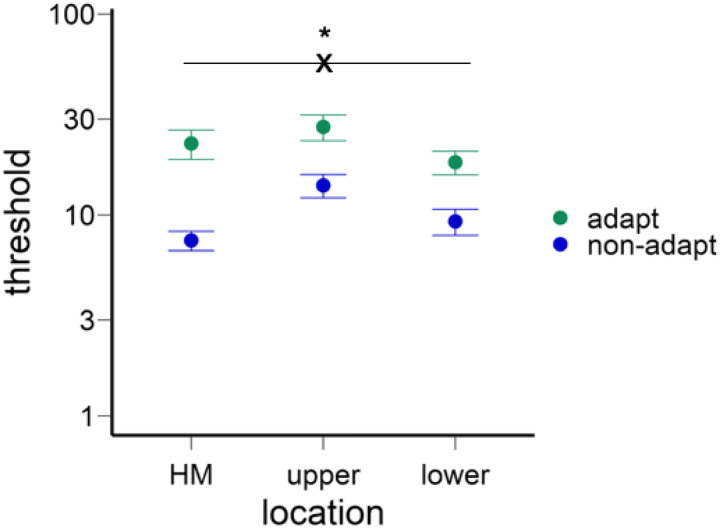
The contrast thresholds for different locations and adaptation conditions. The thresholds were higher in the vertical than horizontal meridian, and higher in the upper than lower vertical meridian. The thresholds were also higher in the adapted and non-adapted conditions. Critically, the adaptation effect was stronger in the horizontal than vertical meridian. The error bars indicate ±1 SEM.

After deriving the c_50_ contrast for the horizontal meridian (HM), upper, and lower vertical meridians for both the adapted and non-adapted conditions, we conducted a two-way ANOVA on contrast thresholds (Figure 3). This analysis showed a main effect of location [*F*(2,22)=7.89, *p*=.003, *η*_*p*_^*2*^=0.42] and a higher threshold in the adapted than non-adapted conditions [*F*(1,11)=18.44, *p*=.001, *η*_*p*_^*2*^=0.63], and an interaction [*F*(2,22)=3.58, *p*=.045, *η*_*p*_^*2*^=0.25], indicating that the adaptation effect varied across locations.

We confirmed that the HVA and VMA emerged in the non-adaptation condition (Figure 3): Contrast thresholds were lower along the horizontal than the vertical meridian [*t*(11)=5.87, *p*<.001, *d*=1.69) and lower at the lower than upper vertical meridian [*t*(11)=2.37, *p*=.037, *d*=0.68].

Next, we assessed the adaptation effect at the horizontal and vertical meridians. The normalized adaptation effect (calculated as the difference between adapted and non-adapted thresholds divided by the sum of the thresholds, as in Lee and Carrasco (2025) was stronger at the horizontal than the vertical meridian [*t*(11)=3.39, *p*=.006, *d*=0.98] (Figure 3, see gaps between adapt and non-adapt conditions for different locations), but no significant difference between the upper and lower vertical meridian [*t*(11)<1].

#### Endogenous attentional effect

**Figure 4.**
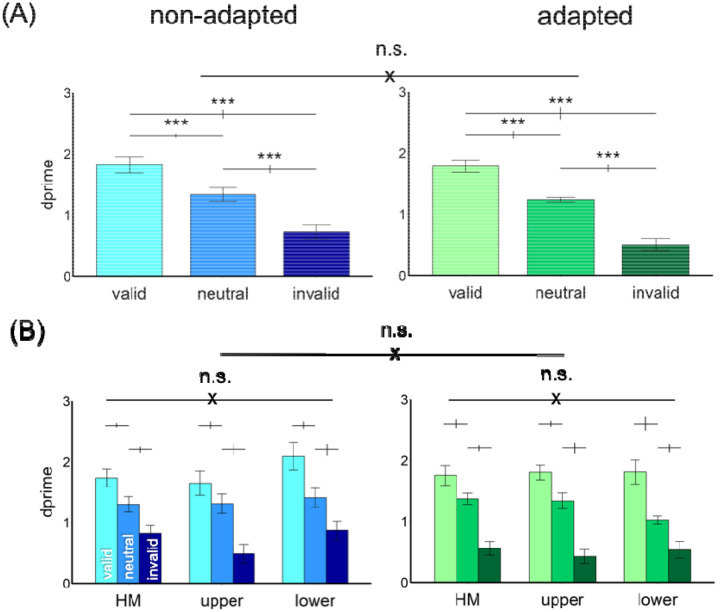
The performance in Experiment 1. (A) d^′^ was higher in the valid followed by neutral and invalid conditions in both non-adapted and adapted conditions. There was no difference between the non-adapted and adapted conditions. (B) The attentional effects were similar around polar angle and were comparable in the non-adapted and adapted conditions. The error bars above the bar plots indicate ±1 SEM of the difference between conditions. *** *p*<.001, n.s. *p*>.05.

Figure 4 shows the results. We compared the endogenous attentional effect on d^′^ by conducting a three-way ANOVA on the factors of location (HM, upper, lower), attentional validity (valid, neutral, invalid), and adaptation (adaptation, non-adaptation). Given that we titrated the contrast thresholds across locations and adaptation conditions, we expected no main effects of either adaptation or location. Indeed, there was a main effect of attention [*F*(2,22)=53.18, *p*<.001, *η*_*p*_^*2*^=0.83], but neither of location [*F*(2,22)<1], nor of adaptation [*F*(1,11)<1]. There was neither a 3-way interaction nor 2-way interactions [all *ps*>.1].

The results were further confirmed by separating the adapted and non-adapted conditions into two 2-way ANOVAs on attention and location. For the non-adapted condition, we observed a main effect of attention [*F*(2,22)=46.74, *p*<.001, *η*_*p*_^*2*^=0.81] but not of location [*F*(2,22)<1] or an interaction [*F*(4,44)=1.68, *p*>.1]. The same pattern emerged for the adapted condition: a main effect of attention [*F*(2,22)=38.59, *p*<.001, *η*_*p*_^*2*^=0.78] but not of location [*F*(2,22)<1] or an interaction [*F*(4,44)=1.48, *p*>.1]. Thus, neither adaptation state nor location modulated the pronounced overall effect of attention.

**Figure 5.**
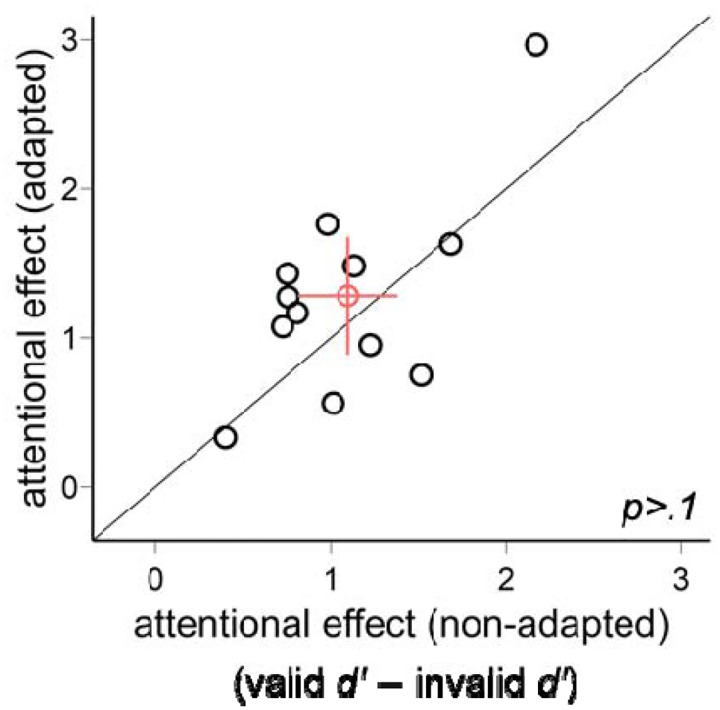
The comparison of the endogenous attentional effects (valid *d*′ − invalid *d*′) in Experiment 1. The attentional effects were comparable in the adapted and non-adapted conditions. The red circle indicates the mean of all participants, and the error bars indicate ±1 SEM.

We plot the individual data for the endogenous attentional effect (valid *d*′ − invalid *d*′) in the adapted and non-adapted conditions (Figure 5). There was no difference between the two conditions [*t*(11)=1.27, *p*>.1].

In sum, the endogenous attentional effect was comparable across locations and adaptation conditions.

## Experiment 2 — Exogenous attention

Experiment 1 shows that endogenous attention does not reshape the performance fields, even after differential adaptation effects across meridians. In Experiment 2, we examined if exogenous attention has a similar or distinct pattern as endogenous attention, given that they have different temporal dynamics and degree of flexibility (e.g., Barbot & Carrasco, 2017; Giordano et al., 2009; Nakayama & Mackeben, 1989; Yeshurun & Carrasco, 1998) and distinct neural underpinnings (Chen et al., 2025; Fernández & Carrasco, 2020; Fernández et al., 2023).

**Figure 6.**
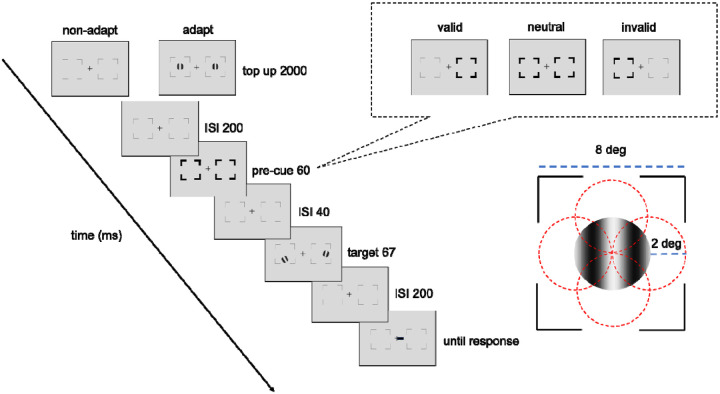
Experimental procedure: The procedure was the same as Experiment 1 except for the pre-cue and ISI timings. The pre-cue (bolded placeholders) either matches (valid condition), mismatches (invalid condition) the response cue, or does not provide location information (neutral condition). The placeholders were wider (8°) than Experiment 1. There were 5 possible target locations, which were 2° away from the central Gabor. For illustration purposes, the stimulus size and spatial frequency shown here are not to scale.

To manipulate exogenous attention, we used a peripheral cue (a bolded placeholder) presented before the target onset. According to the normalization model of attention (Reynolds & Heeger, 2009), exogenous attention can also affect contrast gain when the attentional window is large enough (Herrmann et al., 2010). To induce a large attentional window while maintaining overlap between the target and adaptors to ensure the adaptation effect, we randomly presented the target in one of the five locations within the placeholders (Figure 6), and participants were explicitly instructed to attend to the whole space encompassed by the placeholder, as the target could appear anywhere within the placeholder.

### Methods

#### Participants

Eleven out of 12 participants who participated in Experiment 1, including author HHL, also participated in Experiment 2.

#### Stimuli and apparatus

Figure 6 shows an experimental trial. The target stimuli and the apparatus were the same as Experiment 1. The placeholders in Experiment 2 (length = 0.256° for placeholders that were further away from the center, length = 0.192° for placeholders that were closer to the center, all width = 0.06°) were larger, given that there were 5 possible target locations: center and 2° on the upper, lower, left, or right of the central Gabor. During the cue presentation, the placeholders became thicker (6 pixels bigger for the frame closer to the center and 8 pixels bigger for the frame further away from the center) to capture participants’ exogenous attention.

#### Experimental design and procedures

The same c_50_ contrast derived from Experiment 1 was used in Experiment 2 for the adapted and non-adapted conditions across locations. The experimental design and procedures were the same as in Experiment 1, except for the following: After the top-up, there was a 200-ms ISI before the exogenous pre-cue appeared for 60 ms, followed by 40-ms ISI. The tilted Gabor was then presented for 67 ms followed by another 200-ms ISI and the response cue. The exogenous cues were not informative, 33% of the cues were valid, another 33% were neutral and the other 33% were invalid. Participants were instructed to enlarge their attentional window during the task, as they were explicitly told that the target could appear anywhere within the placeholders.

### Results

**Figure 7.**
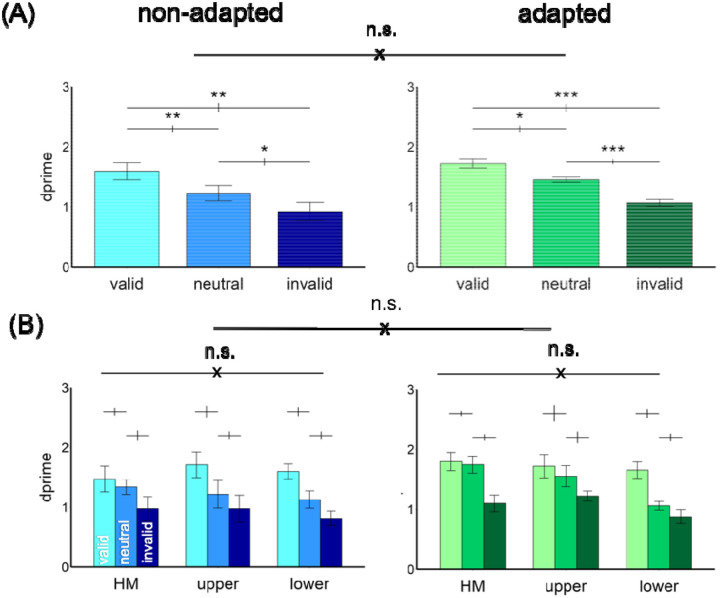
The performance in Experiment 2. (A) *d*′ was higher in the valid followed by neutral and invalid conditions in both non-adapted and adapted conditions. There was no difference between the non-adapted and adapted conditions. (B) The attentional effects were similar around polar angle and were comparable in the non-adapted and adapted conditions. The error bars above the bar plots indicate ±1 SEM of the difference between conditions. *** *p*<.001, ** *p*<.01, * *p*<.05, n.s. *p*>.05.

Figure 7 shows our results. As in Experiment 1, we compared the exogenous attentional effect on d^′^ by conducting a three-way ANOVA on the factors of location (HM, upper, lower), attentional validity (valid, neutral, invalid), and adaptation (adaptation, non-adaptation). There was a main effect of attention [*F*(2,20)=20.7, *p*<.001, *η*_*p*_^*2*^=0.67], but neither of location [*F*(2,20)=2.61, *p*=.099], nor of adaptation [*F*(1,10)=1.08, *p*>.1]. There was neither a 3-way interaction [*F*(4,40)=2.36, *p*=.069] nor 2-way interactions [all *ps*>.1].

The results here were further confirmed by separating the adapted and non-adapted conditions into two 2-way ANOVAs on attention and location. For the non-adapted condition, we observed a main effect of attention [*F*(2,20)=13.33, *p*<.001, *η*_*p*_^*2*^=0.57] but neither of location [*F*(2,20)<1], nor an interaction [*F*(4,40)=1.33, *p*>.1]. The same pattern emerged for the adapted condition: a main effect of attention [*F*(2,20)=21.19, *p*<.001, *η*_*p*_^*2*^=0.68] but neither of location [*F*(2,20)=2.33, *p*>.1] nor an interaction [*F*(4,40)=2.01, *p*>.1].

**Figure 8.**
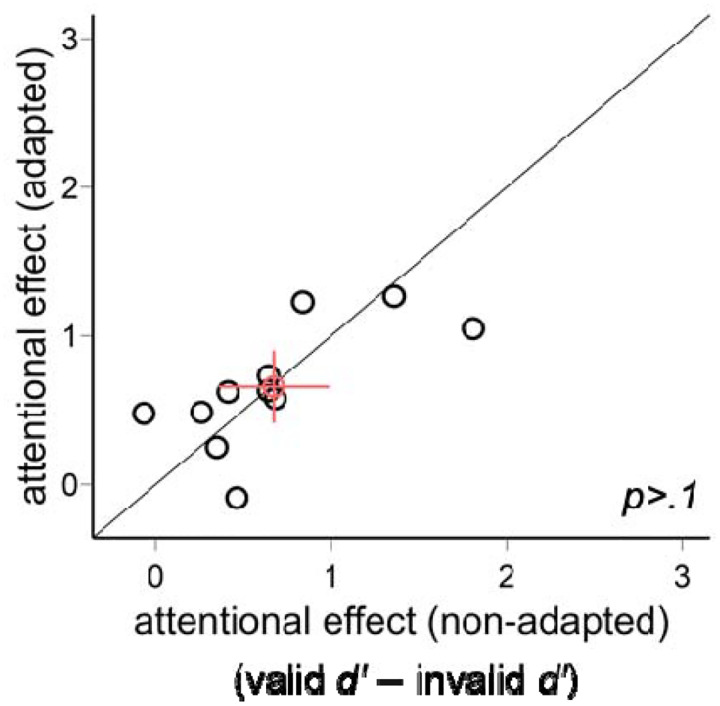
The comparison of the exogenous attentional effects (valid *d*′ − invalid *d*′) in Experiment 2. The attentional effects were comparable in the adapted and non-adapted conditions. The red circle indicates the mean of all participants, and the error bars indicate ±1 SEM.

We plot the individual data for the exogenous attentional effect (valid *d*′ − invalid *d*′) in the adapted and non-adapted conditions (Figure 8). There was no difference between the two conditions [*t*(10)<1].

In sum, the exogenous attentional effect was comparable across locations and adaptation conditions.

## Comparing Experiments 1 and 2

Given that we had 11 common participants in Experiments 1 and 2, we conducted a 4-way repeated-measures ANOVA on the factor of type of attention (endogenous, exogenous), attentional validity (valid, neutral, invalid), adaptation (adapted, non-adapted), and location (HM, upper, lower). There was a main effect of attentional validity [*F*(2,20)=51.72, *p*<.001, *η*_*p*_^*2*^=0.84], and an interaction between attentional validity and type of attention [*F*(2,20)=7.38, *p*=.004, *η*_*p*_^*2*^=0.42]. Post-hoc analyses indicated that valid condition had the highest d’ followed by neutral [valid – neutral: *t*(10)=6.78, *p*<.001, *d*=2.04] and invalid conditions [neutral – invalid: *t*(10)=6.17, *p*<.001, *d*=1.86]. The attentional effect (valid d’ – invalid d’) was stronger for endogenous than exogenous attention [*t*(10)=2.95, *p*=.015, *d*=0.89]. Importantly, there were no 4-way interaction [*F*(4,40)<1] nor any other significant effects [all *ps*>.05], indicating that the effect for both types of attention did not vary across locations nor across adaptation conditions.

Furthermore, we found a positive Pearson correlation [*r*=.39, *p*=.025] between the exogenous and endogenous overall attentional effect (collapsing across adaptation conditions and locations), which indicates that those observers who had a stronger effect of one type of attention also had a stronger effect for the other type.

## Discussion

In this study, we investigated whether attention interacts with adaptation around polar angle. Our results are consistent with separate studies showing: (1) without adaptation, the typical performance fields, with lower contrast thresholds along the horizontal than the vertical meridian (HVA) and along the lower than the upper vertical meridian (VMA) (e.g., Abrams et al., 2012; Baldwin et al., 2012; Cameron et al., 2002; Carrasco et al., 2001; Corbett & Carrasco, 2011; Fuller et al., 2008; Himmelberg et al., 2020; Lee & Carrasco, 2025); (2) stronger adaptation effects at the horizontal than the vertical meridian (Lee & Carrasco, 2025); (3) both endogenous attention (Purokayastha et al., 2021; Tünçok et al., in press) and exogenous attention (Cameron et al., 2002; Carrasco et al., 2001; Roberts et al., 2018; Roberts et al., 2016) enhance contrast sensitivity similarly across all tested locations. Furthermore, our findings revealed that: (1) both types of attention enhance contrast sensitivity at the attended location, with a concomitant decrease at unattended locations; and (2) each endogenous and exogenous attention enhance contrast sensitivity to a similar extent in the adapted and non-adapted conditions –despite differential adaptation effects– and do so uniformly around polar angle.

The finding that endogenous attention enhances contrast sensitivity to a similar extent in adapted and non-adapted conditions, indicates that visual adaptation does not modulate the attention effect. This novel finding is consistent with the corresponding effect of exogenous attention on contrast sensitivity after adaptation (Lee et al., 2024; Pestilli et al., 2007). Despite its flexible nature (reviews: Carrasco, 2011, 2014; Carrasco & Barbot, 2014; Olivers, 2025), endogenous attention neither enhances nor decreases contrast sensitivity differentially before and after adaptation, indicating that these two processes play independent roles in shaping performance.

Typically, the effect of exogenous attention manifests as response gain, and the effect of endogenous attention as contrast gain (Ling & Carrasco, 2006a; Pestilli et al., 2009). In the exogenous attention experiment, we induced contrast gain by manipulating the size of the attentional window, presenting the target Gabor at one of 5 different locations within a larger stimulus placeholder. According to Reynolds and Heeger’s normalization model of attention, attention produces contrast gain rather than response gain when the attentional window is large relative to stimulus size (Reynolds & Heeger, 2009). A psychophysical and fMRI study confirmed these predictions (Herrmann et al., 2010). By contrast, endogenous attention can affect response gain when the attentional window is relatively smaller than the stimulus size (Fernández et al., 2023; Herrmann et al., 2010; Morrone, Denti, & Spinelli, 2004). Consistent with previous findings (Lee et al., 2024; Pestilli et al., 2007), exogenous attentional modulated contrast sensitivity to a similar extent in adapted and non-adapted conditions, indicating that adaptation did not modulate its effect. These results support Hypothesis 1: after adaptation, covert spatial attention modulates contrast sensitivity to the same extent as without adaptation (Figure 1A).

Adaptation was more pronounced at the horizontal than the vertical meridian. Unlike our previous study (Lee & Carrasco, 2025), which blocked each target location, our design introduced higher target uncertainty by using two possible target locations per trial (Figure 2). This indicates that the previous findings were robust to target uncertainty and could be replicated with a different group of participants. Most adaptation studies have examined only the horizontal meridian (e.g., Beaton & Blakemore, 1981; Carrasco et al., 2006; Gao, Webster, & Jiang, 2019; Greenlee, Georgeson, Magnussen, & Harris, 1991; Pestilli et al., 2007; Schieting & Spillmann, 1987), only the vertical meridian (e.g., Bell, Gheorghiu, Hess, & Kingdom, 2011; Bell, Gheorghiu, & Kingdom, 2009; Montaser-Kouhsari & Rajimehr, 2004), or have not analyzed target locations separately (e.g., Bao, Fast, Mesik, & Engel, 2013; Lin, Zhou, Naya, Gardner, & Sun, 2021; Ling & Carrasco, 2006b; Zimmermann, Weidner, Abdollahi, & Fink, 2016). Our results add further evidence that adaptation differs across locations, an important factor to consider in future studies and models of vision.

Endogenous and exogenous attention enhanced contrast sensitivity similarly around polar angle, despite differential adaptation effects. Visual adaptation reduced contrast sensitivity more at the horizontal than vertical meridian, yet neither type of covert spatial attention reshaped the performance fields, i.e., they did not modulate the extent of the asymmetries. This is notable given that endogenous attention is flexible and exogenous attention is automatic (e.g., Carrasco, 2011, 2014; Carrasco & Barbot, 2014; Olivers, 2025), yet both left the performance fields unchanged after adaptation without compensating for poor performance. These findings indicate that visual adaptation does not modulate the effects of covert spatial attention, even at the location of poorest performance, supporting Hypothesis 4 (Figure 1D).

What contributes to performance asymmetries in the HVA and VMA? These asymmetries arise from both retinal and cortical factors. Retinally, cone density is higher at the horizontal than the vertical meridian (Curcio, Sloan Jr, Packer, Hendrickson, & Kalina, 1987; Curcio, Sloan, Kalina, & Hendrickson, 1990), and midget-RGC density is higher at the lower than the upper vertical meridian (Curcio et al., 1990; Song, Chui, Zhong, Elsner, & Burns, 2011). Cortically, the V1 surface area is larger for the horizontal than the vertical meridian, and for the lower than the upper vertical meridian (Benson, Kupers, Barbot, Carrasco, & Winawer, 2021; Himmelberg et al., 2021; Himmelberg, Tünçok, et al., 2023; Himmelberg, Winawer, & Carrasco, 2022, 2023; Lee & Carrasco, 2025; Silva et al., 2018). Moreover, cortical factors account for more variance in these asymmetries than retinal factors (Kupers, Benson, Carrasco, & Winawer, 2022). Still, these factors cannot fully explain the behavioral differences observed in psychophysical tasks (Jigo, Tavdy, Himmelberg, & Carrasco, 2023), suggesting that additional factors –such as sensory tuning and neuronal computations– also contribute to the HVA and VMA (Himmelberg, Winawer, & Carrasco, 2023; Jigo et al., 2023; Xue, Barbot, Abrams, Chen, & Carrasco, 2025).

Endogenous and exogenous attention rely on different neural substrates. Functional magnetic resonance imaging (fMRI) studies show that they differentially modulate activity in the frontoparietal network (Beck & Kastner, 2014; Buschman & Miller, 2009; Chica, Bartolomeo, & Lupiáñez, 2013; Fiebelkorn & Kastner, 2020; Kastner & Buschman, 2017; Meyer, Du, Parks, & Hopfinger, 2018), temporoparietal junction (Dugué, Merriam, Heeger, & Carrasco, 2018), and visual cortex (Dugué, Merriam, Heeger, & Carrasco, 2020; Hopfinger & West, 2006; Ling, Jehee, & Pestilli, 2015). Studies using Transcranial magnetic stimulation (TMS), which disrupts the balance between excitation and inhibition (Bradley, Nydam, Dux, & Mattingley, 2022; Kobayashi & Pascual-Leone, 2003; Valero-Cabré, Pascual-Leone, & Coubard, 2011), further revealed that early visual cortex plays a critical role in adaptation (Lee et al., 2024; Perini et al., 2012) and in exogenous attention (Fernández & Carrasco, 2020; Lee et al., 2024), whereas the human homologue of right frontal eye fields (rFEF+) plays a critical role in endogenous attention (Fernández et al., 2023). Critically, disrupting rFEF+ does not affect exogenous attention (Chen et al., 2025), and disrupting early visual cortex does not affect endogenous attention (Fernández et al., 2023), indicating a double dissociation. Despite these distinct neural underpinnings, the present study shows that both types of covert spatial attention affect contrast sensitivity around polar angle, without interacting with location or adaptation. These findings suggest that distinct neuronal populations modulate polar angle asymmetries, adaptation, and covert spatial attention effects.

We found a stronger attentional effect in the endogenous than exogenous attention. Given that adaptation is more effective when the adaptor and the target spatially overlap (Kovács, Zimmer, Harza, & Vidnyánszky, 2007; Larsson & Harrison, 2015; Webster, 2011, 2015), we manipulated target uncertainty by using 5 possible target locations with 2° of overlap between adaptor and target. This manipulation allowed exogenous attention to enhance contrast sensitivity via contrast gain while still triggering adaptation at the target location. The weaker effect of exogenous than endogenous attention may reflect a narrower attentional window than for endogenous attention in our design, as well as compared with previous studies. For example, Herrmann et al. (2010) used five possible target locations with no overlap, whereas in our current study the target Gabors could overlap by 2° within placeholders. According to the Reynolds and Heeger’s normalization model of attention (Reynolds & Heeger, 2009), attention multiplies stimulus-evoked activity before divisive normalization. In our task, normalization may pool more neighboring neurons in the suppressive drive than with a typical exogenous attention protocol, but not as many as with a typical endogenous attention protocol —leading to less pronounced contrast gain and thus weaker exogenous than endogenous attention effects.

Why do type of spatial covert attention, adaptation, and polar angle asymmetries not interact? The visual cortex plays a crucial role in all three processes. For covert spatial attention, endogenous attention modulates activity in visual cortex via feedback from frontoparietal cortex (Buschman & Miller, 2009; Chica et al., 2013; Corbetta, Patel, & Shulman, 2008; Corbetta & Shulman, 2002; Dugué et al., 2020; Lauritzen, D’Esposito, Heeger, & Silver, 2009; Pestilli, Carrasco, Heeger, & Gardner, 2011), increasingly modulates activity in early visual areas (Dugué et al., 2020), and V1/V2 are not critical for this effect (Fernández et al., 2023). In contrast, exogenous attention modulates visual cortex via feedforward activation (Dassanayake, Michie, & Fulham, 2016; Dugué et al., 2020; Hopfinger, Luck, & Hillyard, 2004; Liu, Pestilli, & Carrasco, 2005; F. Wang, Chen, Yan, Zhaoping, & Li, 2015; Westerberg, Schall, Woodman, & Maier, 2023) and V1/V2 are critical for this effect (Fernández & Carrasco, 2020; Lee et al., 2024). Moreover, these types of attention differentially affect distinct visual subregions of the temporoparietal-junction (Dugué et al., 2018). All these differences underscore the distinct contributions of endogenous and exogenous in modulating visual perception.

The early visual cortex plays a critical role in visual adaptation. Stimulating V1/V2 using TMS decreases the contrast adaptation effect (Perini et al., 2012), and adaptation modulates the contrast response function in V1/V2 (Gardner et al., 2005; Vinke et al., 2022). Although a TMS study revealed that adaptation and exogenous attention interact in early visual cortex (Lee et al., 2024), this does not imply that such interaction varies systematically around polar angle, as other factors can shape asymmetries. Moreover, it is presently unknown whether endogenous attention and adaptation interact in early visual or frontal areas.

Both adaptation (Lee & Carrasco, 2025) and polar angle asymmetries (Himmelberg et al., 2022; Himmelberg, Winawer, & Carrasco, 2023; Lee & Carrasco, 2025) correlate with V1 surface area. Yet surface area cannot fully account for these asymmetries (Jigo et al., 2023); additional factors, such as neural gain also contribute to these asymmetries (Xue et al., 2025). Future research integrating computational modeling, neuroimaging, neurostimulation and psychophysics will be essential to assess the relative contributions of these factors to the differences and commonalities among attention, adaptation and polar angle asymmetries.

In conclusion, this study reveals that performance asymmetries are resistant to the effects of covert spatial attention, both endogenous and exogenous, even after differential adaptation effects across meridians. Although adaptation and attention help us allocate limited resources according to task demands, attention does not differentially enhance target processing at locations of better or worse performance.
